# Development and validation of immediate self-feedback very short answer questions for medical students: practical implementation of generalizability theory to estimate reliability in formative examination designs

**DOI:** 10.1186/s12909-024-05569-x

**Published:** 2024-05-24

**Authors:** Sethapong Lertsakulbunlue, Anupong Kantiwong

**Affiliations:** Department of Pharmacology, Phramongkutklao College of Medicine, Bangkok, 10400 Thailand

**Keywords:** Formative examination, Self-assessment, Immediate feedback, VSAQ, Generalizability theory, Medical Student

## Abstract

**Background:**

Very Short Answer Questions (VSAQs) reduce cueing and simulate better real-clinical practice compared with multiple-choice questions (MCQs). While integrating them into formative exams has potential, addressing marking time and ideal occasions and items is crucial. This study gathers validity evidence of novel immediate self-feedback VSAQ (ISF-VSAQ) format and determines the optimal number of items and occasions for reliable assessment.

**Methods:**

Ninety-four third-year pre-clinical students took two ten-item ISF-VSAQ exams on cardiovascular drugs. Each question comprised two sections: (1) Questions with space for student responses and (2) a list of possible correct answers offering partial-credit scores ranging from 0.00 to 1.00, along with self-marking and self-feedback options to indicate whether they fully, partially, or did not understand the possible answers. Messick’s validity framework guided the collection of validity evidence.

**Results:**

Validity evidence included five sources: (1) Content: The expert reviewed the ISF-VSAQ format, and the question was aligned with a standard examination blueprint. (2) Response process: Before starting, students received an example and guide to the ISF-VSAQ, and the teacher detailed the steps in the initial session to aid self-assessment. Unexpected answers were comprehensively reviewed by experts. (3) Internal structure: The Cronbach alphas are good for both occasions (≥ 0.70). A generalizability study revealed Phi-coefficients of 0.60, 0.71, 0.76, and 0.79 for one to four occasions with ten items, respectively. One occasion requires twenty-five items for acceptable reliability (Phi-coefficient = 0.72). (4) Relations to other variables: Inter-rater reliability between self-marking and teacher is excellent for each item (r_s_(186) = 0.87–0.98,*p* = 0.001). (5) Consequences: Path analysis revealed that the self-reflected understanding score in the second attempt directly affected the final MCQ score (β = 0.25,*p* = 0.033). However, the VSAQ score did not. Regarding perceptions, over 80% of students strongly agreed/agreed that the ISF-VSAQ format enhances problem analysis, presents realistic scenarios, develops knowledge, offers feedback, and supports electronic usability.

**Conclusion:**

Electronic ISF-VSAQs enhanced understanding elevates learning outcomes, rendering them suitable for formative assessments with clinical scenarios. Increasing the number of occasions effectively enhances reliability. While self-marking is reliable and may reduce grading efforts, instructors should review answers to identify common student errors.

**Supplementary Information:**

The online version contains supplementary material available at 10.1186/s12909-024-05569-x.

## Introduction

### VSAQs and its benefits

 Multiple-choice answer Questions (MCQs) with a single best answer are widely used for assessing knowledge in medical education, including national licensing examinations worldwide. Nonetheless, they can also lead to cueing [1], where individuals answer questions based on cues within the question or answer choices rather than relying on their actual content knowledge. Additionally, these MCQs tend to encourage a recognition-based study approach [1, 2]. Furthermore, in real-life clinical scenarios, patients don’t typically present with multiple-choice options, saying, “I have one of these five diagnoses.” Therefore, a Single Best Answer question (SBAQ) format may not fully reflect the complexities of clinical reasoning in real-world settings [3]. In addition, while exams featuring open-ended questions, such as Constructed Response Questions (CRQs) and Modified Essay Questions (MEQs), can assess students’ factual knowledge, they may also have drawbacks, such as being rater-dependent and time-consuming [4].

A relatively new approach, known as Very Short Answer Questions (VSAQs), has emerged as a solution to these issues. An emerging body of evidence has been established supporting the use of VSAQs as an alternative assessment tool in both formative and summative undergraduate evaluations [5–9]. VSAQs are concise, 1–5 words, free-response questions that potentially surpass SBAQs’ effectiveness by discouraging recognition-based study methods and reducing cueing through their open-ended format [2]. Additionally, VSAQs efficiently identify common errors, such as prescribing mistakes and promote open response generation based on student ideas, compared to SBAQs. This makes them valuable for enhancing students’ safe prescribing skills and reducing errors [9, 10].

Despite VSAQs’ potential, recent evidence primarily originates from a limited number of research groups and contexts. It remains uncertain whether implementing VSAQs by less experienced teachers in diverse populations, countries, and medical education settings would yield comparable results [2]. Furthermore, prior studies predominantly concentrated on comparing VSAQs to SBAQs. Although these studies found that VSAQs exhibited superior validity, reliability, and discrimination compared to SBAQs, they did not assess the number of items and occasions needed for a reliable examination [2, 5].

### Utility of self-feedback and VSAQ

Feedback is an integral aspect of the instructional process, offering support and enhancements to learning. Being a fundamental part of both summative and formative assessment, it is not a separate educational entity. Instead, it is an ongoing part of instructional units and assessments [11]. Feedback, as a teaching-learning strategy, has been part of medical education for several decades. However, its global application is often considered suboptimal at best [12]. This may be attributed to the limited number of teachers available to provide feedback to students. Self-assessment through formative examination may solve this problem [13]. It is a vital aspect of the learning process that empowers learners to recognize their learning needs and take the necessary steps to address them [14–16]. Therefore, Phramongkutklao College of Medicine (PCM) developed a novel immediate self-feedback VSAQ (ISF-VSAQ) format, which would assist learners in receiving consistent feedback through formative examinations.

### Messick’s Validity Framework

The study utilized Messick’s validity framework to gather the validity evidence for the ISF-VSAQ [17–19]. This framework offers a systematic approach to collecting construct validity evidence, emphasizing five key aspects: (1) Content, ensuring test items align with the intended construct through blueprints and expert evaluation of preliminary items; (2) Response Process, focusing on data integrity and clear instructions, which necessitates providing clear guidance to participants and thorough training for raters; (3) Internal Structure, reviewing the exam’s psychometric properties, such as Cronbach’s alpha, inter-rater reliability, and generalizability; (4) Relations with Other Variables, exploring theoretical correlations; and (5) Consequences, assessing impacts on learners, instructors, and the overall system [17, 18, 20].

### Generalizability theory

Generalizability theory is a statistical methodology used for evaluating the reliability and is a form of validity evidence of assessment instruments in the field of health professions education. This theory examines various sources of variance, including occasion, item, and student-related factors, providing estimates of the contribution each makes to the overall variability in scores [21]. Additionally, Decision studies can aid in identifying sources of assessment error and inefficiency, offering insights into optimal evaluation formats and scoring criteria [22]. Generalizability theory has been extensively applied in medical education research to determine rating quality and enhance the design and implementation of assessments. It proves particularly valuable in determining the number of items and occasions required to achieve reliable and valid assessments [23].

### Objectives

PCM annually enrolls approximately 100 pre-clinical students, which may constrain the feasibility of providing comprehensive individual feedback, given the limited number of only five pharmacology instructors relative to the student population. Thus, ISF-VSAQs were designed to enhance feedback among the students. This study endeavors to gather the validity evidence of the newly developed ISF-VSAQs formative examination delivered through an electronic platform utilizing Messick’s validity framework, in which a third-year pre-clinical student trials the formative examination during a pharmacology cardiovascular course. Additionally, the study objective is to explore the generalizability of VSAQ scores among medical students across various items and occasions. This would serve as a guide for the optimal number of items and occasions needed for both future VSAQs’ formative and summative examinations. Hence, the adoption of the ISF-VSAQ format has the potential to optimize resource allocation and significantly improve the future implementation of VSAQs.

## Methods

### Content

#### ISF-VSAQ study participants

This is a serial cross-sectional study that analyzes the validity and reliability of two occasions and ten items in the ISF-VSAQ formative examination. This exam is part of a cardiovascular course at PCM in Bangkok, Thailand. The exam was not mandatory, but all students were encouraged to participate in the tests, and 94 out of 95 third-year students (98.9%) participated in both exams. The VSAQ covered various topics on pharmacological drugs, including anti-hypertensive drugs, anti-arrhythmic drugs, anti-anginal drugs, drugs in heart failure, anti-thrombotic drugs, rational drug use, drugs used in dyslipidemia, and drugs used in atherosclerotic cardiovascular disease. The first and second VSAQs are parallel, featuring similar clinical vignettes but different questions. The root mean square deviation (RMSD) parallel index was tested, and all are below 0.50, ranging from 0.07 to 0.43 [24]. All students attended lectures on all these drug groups and engaged in team-based learning on the topic of hypertension and anti-hypertensive drugs.

#### ISF-VSAQs structure

**Fig. 1:**
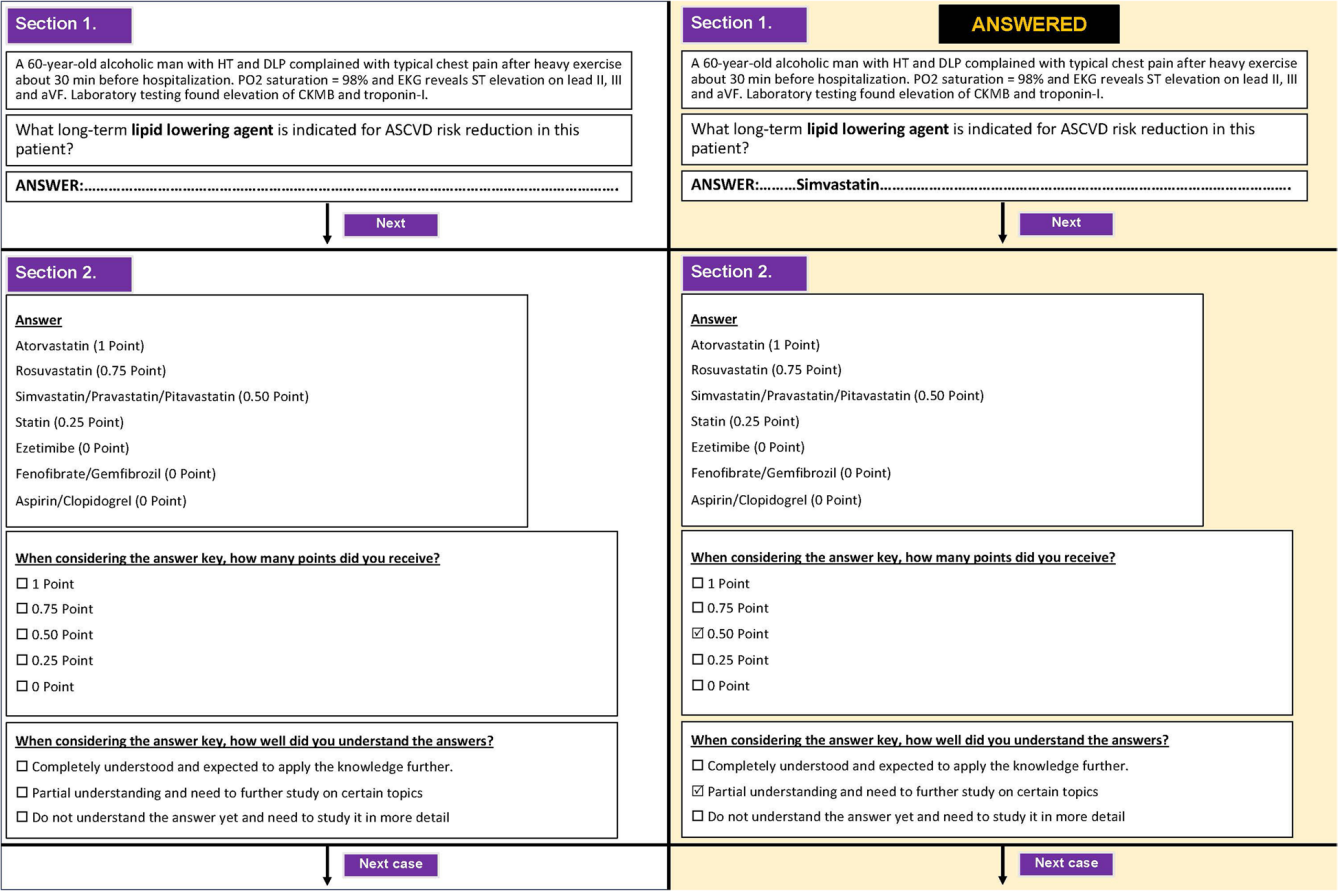
Answered and non-answered immediate self-feedback formative examination formats

Each item of the ISF-VSAQs was structured into two sections with four parts, which included (1) clinical vignettes, a question with space for student response, (2) expected answers with scores and self-scoring choices, and self-feedback on the understanding of the expected answers. The expected answers were graded on a scale of 0.00 to 1.00, with a possible interval of 0.25. The understanding feedback consisted of three levels: (1) Complete Understanding (CU), indicating correct comprehension and an expectation of applying the knowledge further. (2) Partial Understanding (PU), signifying partial comprehension and a need for further study on specific topics. (3) No Understanding (NU), indicating a lack of comprehension of the answer and the need for a more detailed study. The format of the self-feedback formative examination is illustrated in Fig. 1. After completing the VSAQs exam, the students answered five questions regarding their perception of the VSAQs, and they also responded to open-ended questions for further suggestions.

#### Content validity

Three professors validated the VSAQ format through the item-objective congruence (IOC) method. Furthermore, the questions’ content validity was ensured through blueprinting against the third-year curriculum at PCM, ensuring a comprehensive representation of relevant item specifications and alignment with the syllabus. Subsequently, the questions received content validation from three pharmacology professors at PCM.

### Response process

#### Student Preparation and Assessment

A total of 94 third-year pre-clinical medical students took a formative examination comprising 10 ISF-VSAQ format questions via Google Form under exam conditions within one hour. An information sheet about the project was presented on the first page of the Google Form, and participants were asked to review it carefully. Following this, an example question and a guide to the ISF-VSAQ were provided before question number one. Additionally, the teacher carefully explained the steps during the first occasion. The initial formative examination occurred one day after they had completed all the lectures included in the exam. The second formative examination followed a week later, ten days before the final summative examination. Students took between 14 and 36 min for the first attempt and 11 to 31 min for the second attempt. The students were expected to self-rate their VSAQ answers and receive real-time feedback on their scores.

#### Teacher Assessment

The answers were exported into a Microsoft Excel spreadsheet to facilitate teacher ratings of the VSAQ answers. Using the ‘filter’ function in Microsoft Excel, the range of answers for each question was examined, and marks were awarded to answers that matched the expected answers [25]. Minor misspellings or alternative correct spellings were considered correct. Unexpected student answers were subsequently reviewed by three pharmacology professors, who assigned scores. The time required to mark ten items for 94 students was 32 min for the first attempt and 18 min for the second. The longer time for the first attempt was due to the varied answers and the need for professors to validate the scores of unexpected answers.

#### Characteristics and scoring analysis

The data analyses were performed using *IBM SPSS Statistics for Windows, Version 29.0. Armonk, NY: IBM Corp* and *StataCorp, 2021, Stata Statistical Software: Release 17. College Station, TX: StataCorp LLC.* A frequency distribution of demographic characteristics was performed to describe the study subject’s score. Categorical data were presented as percentages, and continuous variables as the median and interquartile range (IQR) or means and standard deviations (SD) as appropriate. A one-way ANOVA was employed to compare the mean scores across three self-feedback understanding score groups separately on the first and second occasions. Levene’s test was conducted to assess the homogeneity of variance, and post-hoc analysis was performed using either the Bonferroni or Dunnett method as deemed appropriate. A Paired-samples t-test was used to compare the difference in means between the first and second VSAQ scores.

### Internal structure

Cronbach’s alpha was utilized to determine the internal consistency reliability of the scores of the ISF-VSAQs. To further find out how reliable the VSAQ score was, a generalizability theory analysis was performed using a three-way ANOVA or a two-faceted, fully crossed random-effect (P×I×O) design. This allowed for a fully crossed design that included people (P), questionnaire items (I), and occasions (O). This analysis estimated various aspects of measurement variance attributed to the facets of the study [22]. Estimate variance components were calculated [26]. It’s important to mention that the analysis examined seven variance components. These included the primary effects of the student’s score (P), items (I), and occasions (O), as well as the two-way interactions between score and items (PI), score and occasions (PO), and item and occasions (IO). Additionally, it accounted for the residual error variance (PRO, e), which captured interactions among all facets and other sources of variability that were not identified. Additionally, a two-facet crossed design decision study examined how the G-coefficient could vary under different facet conditions. The authors calculated the generalizability theory analysis and rechecked it with EduG software [27].

The Phi-coefficient, also known as the absolute G-coefficient, was utilized to assess the reliability of various combinations of facets. This coefficient considers the systematic effects of the facets that could introduce error into the estimate and incorporates it into the error term. The decision to use this absolute coefficient was influenced by the fact that the scores were determined based on predefined criteria rather than relative comparisons. The established minimum threshold for reliability, set at 0.70 for formative and 0.80 for summative examinations, indicates a high degree of generalizability in assessment scores [21, 23, 28].

### Relations to other variables

Criterion-related validity was done by comparing the student-rated scores with teacher ratings. Inter-rater reliability between overall student scores and teacher ratings was calculated using Pearson’s correlation, as the data are continuous and can reasonably be assumed to have a linear relationship. However, for each item, the data violated the normality assumption and was therefore analyzed using Spearman’s correlation. The cutoff for Cronbach’s alpha and correlation is ≥ 0.70 for an acceptable result, ≥ 0.80 for very good, and ≥ 0.90 for excellent outcomes [29].

### Consequences

#### Cut scores for the understanding level, VSAQ and MCQ

Self-feedback understanding was scored as CU = 2, PU = 1, and NU = 0. The summation of the understanding score for each student was stratified into three groups based on tertiles, including ‘Superior Understanding (SU)’ for those in the top tertile, ‘Moderate Understanding (MU)’ for the second tertile, and ‘Inferior Understanding (IU)’ for those in the first tertile. The final pharmacological multiple-choice question (MCQ) exam consisted of 30 questions, with 5 questions each from the following categories: antihypertensive drugs, antiarrhythmic drugs, antianginal drugs, drugs in heart failure, anti-thrombotic drugs, and drugs used in dyslipidemia. Before analyzing their relationship, the MCQ scores on anti-hypertensive drugs were multiplied and weighted in the same proportion as the VSAQ questions. A score above 50% was considered a passing grade for the VSAQs due to the increased difficulty without cues [5, 25], while 60% was set as the passing threshold for the final MCQ, aligning with PCM’s passing standards.

#### Relationship analysis between understanding Level, VSAQ and final MCQ examination

Chi-square tests were employed to assess the relationship between the pass or fail outcomes of the VSAQs and the final MCQs, which were divided into two categories. Cramér’s V was used to determine the association between the tertiles of self-reflected understanding levels, which had three categories and passing the final MCQ.

Path analysis was conducted using *StataCorp, 2021, Stata Statistical Software: Release 17. College Station, TX: StataCorp LLC*, with maximum likelihood extraction, to investigate the relationship between passing the VSAQ exam, understanding level, and the student’s final pharmacological MCQ exam scores. The model’s goodness of fit was evaluated using six indices: (1) the chi-square test, χ²; (2) the chi-square test over degrees of freedom (df), χ²/df; (3) the Comparative Fit Index (CFI); (4) the Tucker-Lewis Index (TLI); (5) the Root Mean Square Error of Approximation (RMSEA); and (6) the Standardized Root Mean Square Residual (SRMR). All of these indices indicated a good fit for the model. A χ²/df ratio less than 2, CFI and TLI values greater than 0.95, and RMSEA and SRMR values less than 0.05 suggested a strong fit between the data and the hypothesized model [30, 31].

#### Student’s perception and suggestions toward ISF-VSAQ

Students’ perceptions toward ISF-VSAQ were gathered after the formative examination, which consisted of five 5-point Likert scale questions. These questions assessed whether the ISF-VSAQ effectively improved knowledge review and problem analysis, aligned with real situations and critical thinking skills, helped in identifying areas for improvement, aided in comprehensive knowledge development, provided useful assessment feedback, and facilitated easy practice of problem-solving on electronic platforms. Following the Likert scale questions, an open-ended question solicited suggestions regarding the ISF-VSAQ. Content analysis was then employed to analyze the students’ suggestions regarding the content of the ISF-VSAQs.

## Results

### Content

Based on the IOC appraised by three experts, all items scored above 0.50, ranging from 0.67 to 1.00. The majority of comments and changes were made to the possible answers to the questions. For example, in the item provided in Fig. 1, the experts suggested adding different types of statins apart from those listed in the National List of Essential Drugs and gave partial scores as appropriate.

### Response process

#### Characteristics of participants’ VSAQs scores and understanding levels

Ninety-four third-year pre-clinical medical students in PCM participated in the formative examination. The average VSAQ score stratified by the self-reflected understanding scale was demonstrated in Table 1. The overall average score in the first attempt was highest in the SU group (6.18 ± 1.95), followed by a gradual decrease in the MU group (4.98 ± 1.44) and the IU group (2.71 ± 1.64), respectively (F(2, 91) = 35.03, *p* = 0.001, η^2^ = 0.44). Similarly, in the second attempt, the VSAQs score was highest among the SU group (7.36 ± 1.73), with a gradual decrease observed in the MU group (5.84 ± 1.34) and the IU group (3.66 ± 1.94), respectively (F(2, 91) = 36.63, *p* = 0.001, η^2^ = 0.45). The average score for each item is also highest in the CU group and decreases in the MU and NU groups, respectively. The average understanding score for each understanding group stratified by attempt is depicted in Supplementary Fig. 1. Furthermore, the overall understanding score increased from 9.64 ± 4.44 in the first attempt to 10.72 ± 4.68 in the second attempt (t(93) = 2.87, *p* = 0.005), *d* = 0.24.

**Table 1:** Average VSAQs score stratified by self-reflected understanding scale

Item	VSAQs attempt	Mean ± SD			Statistical testings				Post-hoc analysis	*p*-value
		CU	PU	NU	Levene Statistic	*p*-value	F	*p*-value		
Item 1	first	0.93 ± 0.18	0.51 ± 0.40	0.03 ± 0.12	16.447	0.001	44.779	0.001	NU < PU	0.001
									NU < CU	0.001
									PU < CU	0.001
	second	0.92 ± 0.21	0.80 ± 0.33	0.03 ± 0.12	13.128	0.001	81.735	0.001	NU < PU	0.001
									NU < CU	0.001
Item 2	first	0.86 ± 0.26	0.60 ± 0.38	0.21 ± 0.40	6.370	0.003	13.054	0.001	NU < CU	0.025
									PU < CU	0.001
	second	0.97 ± 0.15	0.75 ± 0.33	0.06 ± 0.18	30.398	0.001	62.396	0.001	NU < PU	0.001
									NU < CU	0.001
									PU < CU	0.004
Item 3	first	0.71 ± 0.31	0.54 ± 0.22	0.04 ± 0.29	3.631	0.030	45.080	0.001	NU < PU	0.001
									NU < CU	0.001
	second	0.88 ± 0.29	0.40 ± 0.51	0.04 ± 0.35	6.035	0.003	30.243	0.001	NU < PU	0.008
									NU < CU	0.001
									PU < CU	0.001
Item 4	first	0.91 ± 0.27	0.67 ± 0.36	0.12 ± 0.21	6.765	0.002	56.678	0.001	NU < PU	0.001
									NU < CU	0.001
									PU < CU	0.009
	second	0.91 ± 0.22	0.56 ± 0.36	0.16 ± 0.3	5.968	0.004	44.156	0.001	NU < PU	0.001
									NU < CU	0.001
									PU < CU	0.001
Item 5	first	0.69 ± 0.48	0.38 ± 0.35	0.07 ± 0.14	33.180	0.001	27.028	0.001	NU < PU	0.001
									NU < CU	0.001
	second	0.88 ± 0.34	0.62 ± 0.43	0.06 ± 0.19	20.384	0.001	59.062	0.001	NU < PU	0.001
									NU < CU	0.001
Item 6	first	0.83 ± 0.35	0.51 ± 0.34	0.06 ± 0.16	3.680	0.029	36.839	0.001	NU < PU	0.001
									NU < CU	0.001
									PU < CU	0.001
	second	0.89 ± 0.30	0.63 ± 0.31	0.08 ± 0.28	1.708	0.187	37.123	0.001	NU < PU	0.001
									NU < CU	0.001
									PU < CU	0.001
Item 7	first	0.59 ± 0.44	0.43 ± 0.39	0.02 ± 0.06	105.583	0.001	36.564	0.001	NU < PU	0.001
									NU < CU	0.004
	second	0.70 ± 0.38	0.42 ± 0.34	0.06 ± 0.17	18.329	0.001	31.412	0.001	NU < PU	0.001
									NU < CU	0.001
Item 8	first	0.94 ± 0.21	0.55 ± 0.33	0.14 ± 0.22	4.941	0.009	63.169	0.001	NU < PU	0.001
									NU < CU	0.001
									PU < CU	0.001
	second	0.85 ± 0.33	0.44 ± 0.35	0.09 ± 0.20	7.328	0.001	36.870	0.001	NU < PU	0.001
									NU < CU	0.001
									PU < CU	0.001
Item 9	first	0.72 ± 0.38	0.46 ± 0.30	0.03 ± 0.09	22.855	0.001	54.635	0.001	NU < PU	0.001
									NU < CU	0.001
									PU < CU	0.032
	second	0.90 ± 0.29	0.45 ± 0.27	0.10 ± 0.22	0.187	0.830	64.762	0.001	NU < PU	0.001
									NU < CU	0.001
									PU < CU	0.001
Item 10	first	0.63 ± 0.42	0.44 ± 0.24	0.17 ± 0.24	8.733	0.001	13.917	0.001	NU < PU	0.001
									NU < CU	0.010
	second	0.98 ± 0.15	0.87 ± 0.33	0.13 ± 0.34	9.192	0.001	84.611	0.001	NU < PU	0.001
									NU < CU	0.001
Total	first	6.18 ± 1.95^a^	4.98 ± 1.44^b^	2.71 ± 1.64^c^	0.928	0.399	35.030	0.001	c < a	0.001
									c < b	0.001
									b < a	0.021
	second	7.36 ± 1.73^a^	5.84 ± 1.34^b^	3.66 ± 1.94^c^	2.770	0.068	36.630	0.001	c < a	0.001
									c < b	0.001
									b < a	0.002

*CU* complete understanding; Understand correctly and expect to apply the knowledge further, *PU* partial understanding; Understand partially and need to further study on certain topics, *NU* no understanding; Do not understand the answer yet and need to study it in more detail; ^a^Superior understanding is the sum of understanding score in the third tertile; ^b^Moderate understanding is the sum of understanding score in the second tertile; ^c^Inferior understanding is the sum of understanding score in the first tertile

### Internal structure

#### Internal consistency reliability and generalizability study

The overall Cronbach’s alpha for the first and second VSAQ is 0.75 (95% CI: 0.68 to 0.82) and 0.72 (95% CI: 0.64 to 0.80), respectively. Table 2 presents the results of the two-facet Generalizability study for P×I×O designs for the VSAQs exam. The findings reveal that the percentage of variance attributable to the universe score, students (P), is 16.47%, and items (I) account for 9.98% of the total variance. The percentage of variance in the interaction between students and items is 10.98%, while occasions (O) contribute only 2.10%, with a higher contribution from the residuals (55.38%).

**Table 2:** Generalizability study for P×I×O for immediate self-feedback VSAQs formative examination of cardiovascular drugs, among 94 pre-clinical medical students, 10 items and 2 occasions

**Source of Variation P×I×O design**	***df***	**SS**	**MS**	**Estimated Variance Component**	**% of Total Variance**
**Student (P)**	93	78.962	0.849	0.033	16.47
**Item (I)**	9	41.422	4.602	0.020	9.98
**Occasion (O)**	1	4.750	4.750	0.004	2.10
**PI**	837	129.596	0.155	0.022	10.98
**PO**	93	12.944	0.139	0.003	1.50
**IO**	9	7.114	0.790	0.007	3.60
**Residual (PIO, e)**	837	93.067	0.111	0.111	55.38
**Total**	1879	367.856		0.200	100.00

*SS* Sum of squares, *MS* Mean of squares, *df* Degree of freedom

#### Decision study

**Fig. 2:**
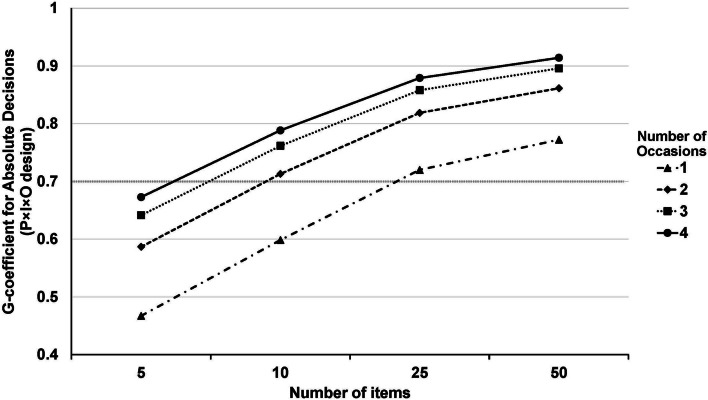
Decision study results for the pre-clinical medical students (*n* = 94) taking Very Short Answer Questions (VSAQs) exams on two occasions, each with ten items. The coefficients represent the projected Phi-coefficient for various combinations of items and occasions. The dotted line indicates an acceptable reliability of above 0.70

The Decision Study of the P×I×O design is shown in Supplementary Table 1. The table displays the Phi-coefficient, which, for one occasion, ranges from 0.47 to 0.77 across five to fifty items. For a single occasion, at least twenty-five items (Phi-coefficient = 0.72) are necessary for a reliable assessment. For two occasions, the Phi-coefficient ranges from 0.59 to 0.86, and only ten items (Phi-coefficient = 0.71) are sufficient for reliable assessment. For three occasions, the Phi-coefficient spans from 0.64 to 0.90; for four occasions, it ranges from 0.67 to 0.91. Figure 2 presents the Phi-coefficient for the absolute decision for the P×I×O designs.

### Relations to other variables

#### Inter-rater reliability between self and teacher-rated

**Fig. 3:**
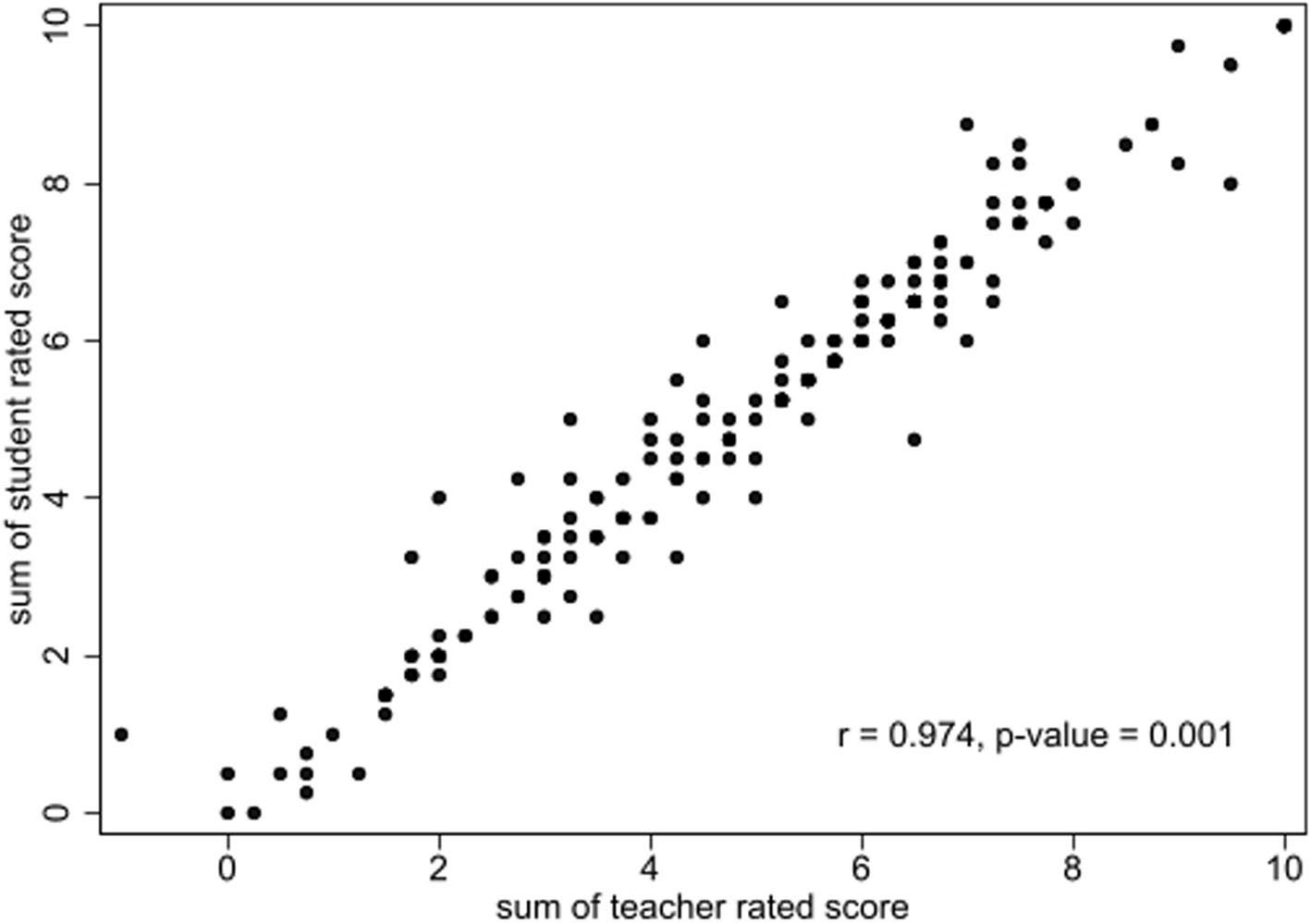
Scatter plot depicting the average student and teacher-rated scores

The inter-rater reliability comparison between self-student-rated and teacher-rated scores is shown in Table 3. The mean scores are 5.03 ± 2.27 and 4.92 ± 2.30 for student and teacher ratings, respectively. For each item, Spearman’s Rho correlations range between 0.87 and 0.98, with an overall Pearson’s correlation of r(186) = 0.97, *p* = 0.001. Figure 3 reveals a scatter plot of the overall scores rated by students and teachers.

**Table 3:** Comparison of Inter-rater reliability between student and teacher for immediate self-feedback VSAQs examination

**Item**	**Student rated**	**Teacher rated**	**Spearman’s** **Rho**	***p*-value**
	**mean±SD**	**mean±SD**		
**Q1. Antihypertensive drugs**	0.63±0.43	0.63±0.44	0.96	0.001
**Q2. Antihypertensive drugs**	0.76±0.36	0.77±0.37	0.96	0.001
**Q3. Antihypertensive drugs**	0.44±0.52	0.45±0.48	0.97	0.001
**Q4. Antiarrhythmic drugs**	0.58±0.43	0.59±0.43	0.97	0.001
**Q5. Drugs used in heart failure**	0.32±0.42	0.30±0.42	0.87	0.001
**Q6. Antianginal drugs**	0.62±0.42	0.57±0.41	0.88	0.001
**Q7. Antithrombotic drugs**	0.25±0.36	0.24±0.35	0.95	0.001
**Q8. Drugs used in dyslipidemia**	0.48±0.41	0.49±0.42	0.92	0.001
**Q9. CVS rational drug used**	0.39±0.39	0.33±0.36	0.89	0.001
**Q10. Drugs used in ASCVD**	0.57±0.41	0.56±0.43	0.98	0.001
**Total**	5.03±2.27	4.92±2.30	0.97*	0.001*

*SD* Standard deviation, *CVS* Cardiovascular system, *ASCVD* Atherosclerotic cardiovascular disease, *Pearson’s correlation was used to analyze the total score

### Consequences

#### Relationship between participants’ VSAQs scores, understanding levels, and pharmacological MCQ scores

Supplementary Fig. 2 illustrates the relationship between passing VSAQs, understanding level groups, and passing the MCQ. As expected, individuals who passed the first VSAQ had a significant relationship with passing the second VSAQ (χ^2^(1, *N* = 94) = 13.990, *p* = 0.001). However, those who do or do not pass the VSAQ do not show a significant difference in passing the final MCQ. When stratified by understanding tertile, the proportion of those who pass the final MCQ does not differ significantly.

**Fig. 4:**
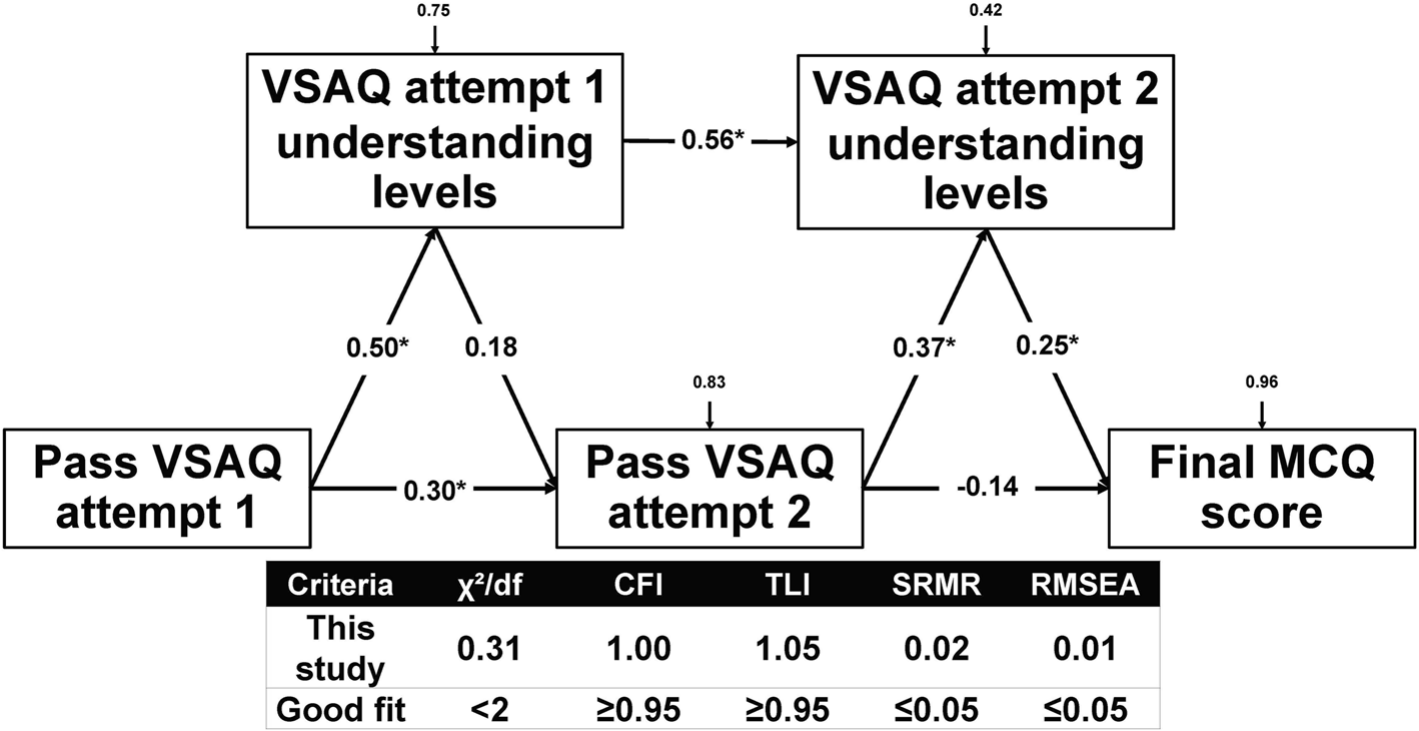
The path analysis of passing Immediate Self-feedback Very Short Answer Questions (ISF-VSAQs), understanding levels, and final multiple-choice question (MCQ) scores. *p < 0.05

Figure 4 shows the path analysis depicting the relationship between participants’ VSAQ scores, understanding levels, and pharmacological MCQ scores. The goodness of fit test resulted in a normed Chi-square value (χ²/df) of 0.31, CFI = 1.00, TLI = 1.05, RMSEA = 0.01, and SRMR = 0.02, indicating a good fit for the data. The path analysis was constructed using five observed variables, with the final MCQ score as the primary outcome. The results are further depicted in Supplementary Table 2. Passing the first VSAQ demonstrates a strong direct effect on VSAQ understanding levels (β = 0.50, *p* = 0.001) and passing the second VSAQ (β = 0.30, *p* = 0.004). Passing the first VSAQ also has indirect effects on the second VSAQ understanding (β = 0.39). The understanding level of the first VSAQ attempt also has a direct effect on the understanding level of the second attempt (β = 0.56, *p* = 0.001). Surprisingly, passing the second VSAQ has a total effect − 0.05 on the MCQ score. However, the second VSAQ understanding level has a significant direct effect on the MCQ score (β = 0.25, *p* = 0.033).

#### Perceptions and suggestions

**Fig. 5:**
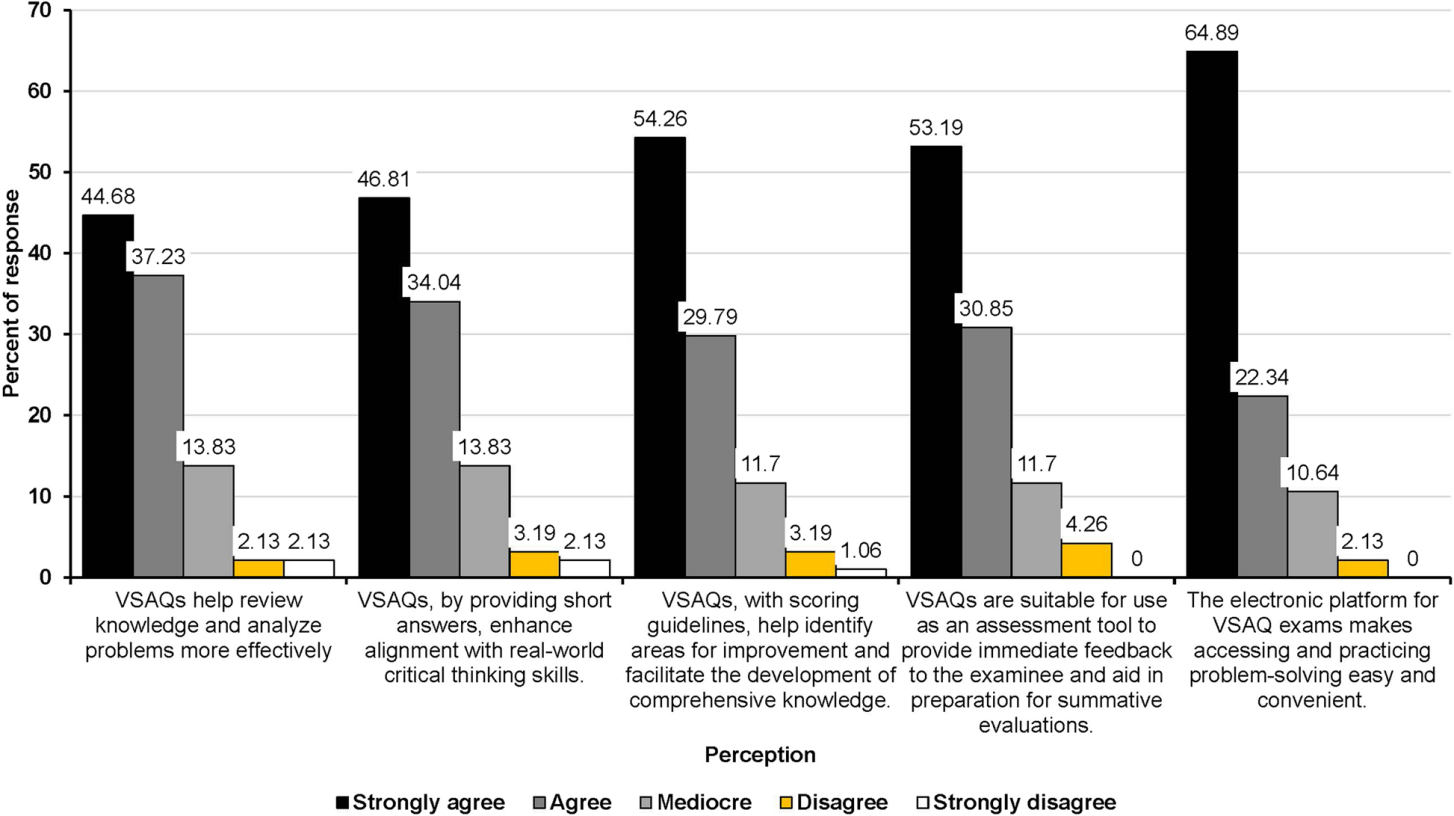
Students’ perception of immediate self-feedback Very Short Answer Questions (ISF-VSAQs) exams

More than 80% of students strongly agreed or agreed that VSAQs effectively improve knowledge review and problem analysis (81.91%), align with real situation and critical thinking skills (80.85%), find areas for improvement (84.05%), help in comprehensive knowledge development (84.25%), give useful assessment feedback (84.04%), and make it easy to practice solving problems on electronic platform (87.15%) (Fig. 5). The overall mean perception score was 21.56 ± 3.72 out of 25. The Cronbach’s alpha for the questionnaire is 0.90 (95%CI: 0.88 to 0.93). From the suggestions, eight students acknowledged that immediate feedback clearly points out their lack of knowledge, encouraging them to review specific topics they missed. On the other hand, five students wished for an immediate explanation of the answers after they had answered the questions.

## Discussion

The current study introduced a novel VSAQ approach with an immediate self-feedback format among 94 third-year pre-clinical students, using Messick’s validity framework to gather evidence. This framework gathered validity evidence of the ISF-VSAQ format’s development from the preparation phase to the outcomes. Furthermore, the optimal number of items and occasions was successfully determined based on the generalizability study.

The generalizability study in the current research revealed that the percentage variance of the subject under measurement is comparable to related studies conducted about students’ self- and peer-assessment [32, 33]. Previous studies have also shown high reliability in self-assessment for formative examinations [33, 34]. Nevertheless, the residual variance remains high, suggesting that potential factors are still unexplored, such as the different types of settings and the number of possible answers and their components.

This study determined that using VSAQs in formative examinations requires at least one occasion with twenty-five items. On two occasions, only ten items are required. Therefore, if only one feedback occasion is available, twenty-five VSAQ items might be reliable. However, multiple occasions of formative VSAQ examinations have been shown to enhance summative assessment outcomes and knowledge retention among students [35]. Consequently, the current study recommends implementing multiple occasions with at least two formative VSAQ examinations.

However, even though using only twenty-five items might achieve acceptable reliability on one occasion, the time estimated for students to complete a ten-item immediate self-feedback VSAQ successfully is approximately 30 to 60 min. Therefore, increasing the number of items beyond twenty-five raises concerns about potentially lengthening the examination period for formative purposes. Additionally, self-feedback quality might decline, as too many questions could lead to cognitive overload.

Despite wide evidence supporting the benefits of self-assessment in gaining better insights into one’s knowledge and motivating students to learn, the use of self- and peer-assessment raises concerns regarding its validity, particularly when comparing students’ ratings to those of teachers as the gold standard [34, 36]. In the current study, the inter-rater reliability between teachers and self-ratings is excellent, likely because students can easily compare their answers with the expected responses and scores. This inter-rater reliability is relatively higher than previous meta-analysis focused on self- and peer-assessment, which typically had an average reliability of 0.63 [34].

Discrepancies in this study likely arose from misconceptions. For example, students sometimes misidentified their incorrect answers as correct due to similarities in wording components, thus awarding themselves full marks. Additionally, for questions with two components, some students noted only one component but still rated themselves full marks. However, after reviewing these and providing feedback, the teacher was able to clarify these misunderstandings, incorporate the incorrect answers into the pool of possible answers, and encourage students to improve their self-assessment skills, a vital aspect of medical education [37].

Most of the student feedback indicated that VSAQs effectively enhance knowledge, align with critical thinking skills, help identify areas for improvement, facilitate comprehensive knowledge development, and provide valuable assessment feedback. Moreover, the students perceived the electronic platform positively, finding it convenient. The electronic platform is also easier to mark as it allows for export as an Excel file. Therefore, the electronic platform is preferred over the paper-based format. However, some students did not comprehend the purpose of receiving only the answer without an explanation, and as a result, they disagreed with the effectiveness of VSAQs.

The decision to provide only the answer was intended to motivate students to independently investigate why their responses received partial scores and why different marks were assigned to each expected answer. This discrepancy in understanding may be attributed to the fact that some students still lean towards teacher-centered education rather than self-driven learning, particularly those concerned about time management [38, 39]. Therefore, further investigation into the perception of self-directed learning and its connection with the preference for self-feedback VSAQs may be necessary.

This study’s path analysis highlights the significance of understanding levels, which more strongly influence final MCQ scores than VSAQ scores. The findings suggest that gaining insights into the answers is more crucial than providing correct responses during formative assessments. Consequently, this underscores the value of multiple formative evaluations in enhancing students’ insights prior to summative assessments [40]. Results align with previous studies using certainty-based marking, which assesses students’ confidence during formative exams and encourages self-reflection and detailed feedback [40, 41]. Nevertheless, further research is needed to determine the optimal number of sessions for maximizing knowledge retention with the ISF-VSAQ.

The advantage of VSAQ as a feedback tool is its potential to offer better assessments than SBAQ [5]. The presence of multiple possible answers encourages students to explore not only the best treatment or management of the clinical vignette but also alternative treatment options. However, despite the great potential of VSAQ, concerns have been raised about the resources and additional time required for marking compared to SBAQ [6]. Nevertheless, the current study demonstrates that self-marking has excellent inter-rater reliability compared to teacher-rated assessments, which helps address the marking issue.

In developing effective self-marking tools, it is imperative to provide a clear and concise guide that is accessible even to students lacking assessment experience [26, 37]. It is recommended that explanations and examples be provided prior to engagement with the activity [42]. Moreover, the validation process of students’ self-marked VSAQ responses by the teachers in charge should be done after every ISF-VSAQ session to accommodate unforeseen answers and furnish students with feedback, thereby fortifying the robustness of self-assessment for future iterations [5, 25].

The study has several strengths. To our knowledge, this is the first study to introduce an ISF-VSAQ format among medical students. Robust validity evidence was gathered utilizing Messick’s validity framework. Generalizability theory analysis was also employed to assess the VSAQ scores and determine the optimal number of items and occasions. This format can facilitate individualized feedback for students in addition to feedback from teachers. Thus, the application of the ISF-VSAQ format in different subject contexts and with various participant groups in future research was encouraged.

The present study has some limitations that need to be acknowledged. Firstly, the study sample only included third-year pre-clinical students in a specific educational setting (i.e., PCM). Therefore, further research is needed to investigate the generalizability of the study findings across different educational settings and multiple study years, as well as the clinical environment and different cultures. Secondly, the study collected only the VSAQs’ performance among the study population, and no comparison tests were done. Therefore, future controlled trials might be needed to assess the effectiveness of the VSAQ format. Finally, because the format is relatively new, some students may not be accustomed to the formative examination. However, the students were guided on the steps to complete the VSAQs, and examples of the questions and the purpose of the understanding feedback section were provided before the exam. Furthermore, the cutoff points of 50% were roughly estimated and may need adjustment using different approaches, such as the Modified Angoff or Ebel method, to establish standards for future courses.

## Conclusion

This study reviewed the validity evidence of the newly developed ISF-VSAQ format. The potential of integrating VSAQs into formative examinations has been previously demonstrated. However, certain limitations regarding marking time and the optimal number of occasions and items have yet to be assessed. Self-marking in formative exams exhibited excellent inter-rater reliability and minimized the limitations associated with prolonged marking time for VSAQs. Furthermore, this study provides evidence that two occasions are necessary to achieve acceptable reliability with a ten-item VSAQ examination. The self-reflected understanding level was also shown to be related to the MCQ score. Consequently, teachers are encouraged to analyze common errors made by students and provide guidance before the summative examination.

### Supplementary Information

Supplementary Material 1.

## Data Availability

The datasets used and/or analyzed during the current study are available by reasonable request from the author via Sethapong.ler@pcm.ac.th.

## Abbreviations

VSAQ: Very short answer question
MCQ: Multiple-choice question
SU: Superior understanding
MU: Moderate understanding
IU: Inferior understanding
CRQ: Constructed response question
MEQ: Modified essay question
G-theory: Generalizability theory
D-study: Decision study
PCM: Phramongkutklao college of medicine
CU: Complete understanding
PU: Partial understanding
NU: No understanding
