# Supplementary material for: Development and validation of immediate self-feedback very short answer questions for medical students: practical implementation of generalizability theory to estimate reliability in formative examination designs
Source: BMC Med Educ. 2024 May 24;24:572. doi: 10.1186/s12909-024-05569-x (PMC11127299; doi:10.1186/s12909-024-05569-x)

**Supplementary File**

**Development and Validation of Immediate Self-Feedback Very Short Answer Questions for Medical Students: Practical Implementation of Generalizability Theory to Estimate Reliability in Formative Examination Designs**

Sethapong Lertsakulbunlue^1^,*Anupong Kantiwong^1^

^1^Department of Pharmacology, Phramongkutklao College of Medicine, Bangkok 10400, Thailand

| **Supplementary Figure 1.** Average immediate self-feedback VSAQs score stratified by self-reflected understanding scale | Page 2 |
| --- | --- |
| **Supplementary Table 1.** Decision Study of P×I×O design for ISF-VSAQs formative assessment of cardiovascular drugs | Page 3 |
| **Supplementary Table 2.** Path analysis of passing VSAQ, VSAQ understanding levels, and pharmacological MCQ scores | Page 4 |
| **Supplementary Figure 2.** The relationship between passing ISF-VSAQs, understanding level groups, and passing the final MCQ exam. | Page 5 |

**Supplementary Figure 1.** Average understanding scores for each understanding group, stratified by attempt. Superior Understanding for those in the top tertile, Moderate Understanding for the second tertile, and Inferior Understanding for those in the first tertile.


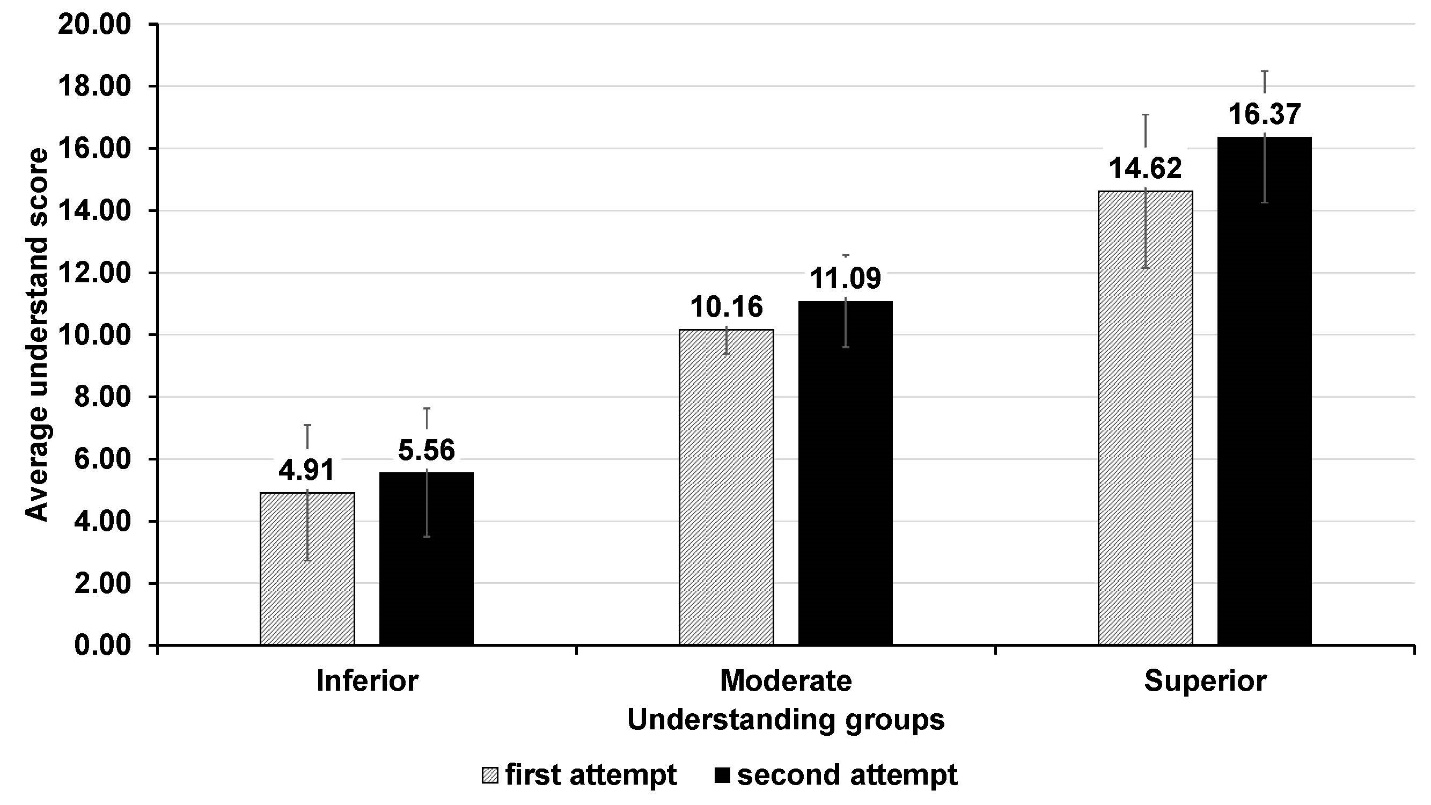


| **Supplementary Table 1.** Decision Study of P×I×O design for ISF-VSAQs formative assessment of cardiovascular drugs | | | | | | | | | | | | | | | | | |
| --- | --- | --- | --- | --- | --- | --- | --- | --- | --- | --- | --- | --- | --- | --- | --- | --- | --- |
| **Effect** | **Estimate variance components in D-Study** | | | | | | | | | | | | | | | | |
|  | **n_o_'** | **1** | **1** | **1** | **1** | **2** | **2** | **2** | **2** | **3** | **3** | **3** | **3** | **4** | **4** | **4** | **4** |
|  | **n_i_'** | **5** | **10** | **25** | **50** | **5** | **10** | **25** | **50** | **5** | **10** | **25** | **50** | **5** | **10** | **25** | **50** |
| σ_p_^2^ |  | 0.032 | 0.032 | 0.032 | 0.032 | 0.032 | 0.032 | 0.032 | 0.032 | 0.032 | 0.032 | 0.032 | 0.032 | 0.032 | 0.032 | 0.032 | 0.032 |
| σ_i_^2^ |  | 0.004 | 0.002 | 0.001 | 0.000 | 0.004 | 0.002 | 0.001 | 0.000 | 0.004 | 0.002 | 0.001 | 0.000 | 0.004 | 0.002 | 0.001 | 0.000 |
| σ_o_^2^ |  | 0.004 | 0.004 | 0.004 | 0.004 | 0.002 | 0.002 | 0.002 | 0.002 | 0.001 | 0.001 | 0.001 | 0.001 | 0.001 | 0.001 | 0.001 | 0.001 |
| σ_pi_^2^ |  | 0.005 | 0.002 | 0.001 | 0.000 | 0.005 | 0.002 | 0.001 | 0.000 | 0.005 | 0.002 | 0.001 | 0.000 | 0.005 | 0.002 | 0.001 | 0.000 |
| σ_po_^2^ |  | 0.003 | 0.003 | 0.003 | 0.003 | 0.001 | 0.001 | 0.001 | 0.001 | 0.001 | 0.001 | 0.001 | 0.001 | 0.001 | 0.001 | 0.001 | 0.001 |
| σ_io_^2^ |  | 0.001 | 0.001 | 0.000 | 0.000 | 0.001 | 0.000 | 0.000 | 0.000 | 0.000 | 0.000 | 0.000 | 0.000 | 0.000 | 0.000 | 0.000 | 0.000 |
| σ_pio_^2^ |  | 0.020 | 0.010 | 0.004 | 0.002 | 0.010 | 0.005 | 0.002 | 0.001 | 0.007 | 0.003 | 0.001 | 0.001 | 0.005 | 0.003 | 0.001 | 0.001 |
| σ̂^2^_δ_ | | 0.027 | 0.015 | 0.008 | 0.005 | 0.016 | 0.009 | 0.004 | 0.003 | 0.012 | 0.007 | 0.003 | 0.002 | 0.010 | 0.006 | 0.003 | 0.002 |
| σ̂^2^_Δ_ | | 0.036 | 0.021 | 0.012 | 0.009 | 0.023 | 0.013 | 0.007 | 0.005 | 0.018 | 0.010 | 0.005 | 0.004 | 0.016 | 0.009 | 0.004 | 0.003 |
| E*ρ*^2^ | | 0.538 | 0.680 | 0.806 | 0.860 | 0.666 | 0.786 | 0.881 | 0.919 | 0.723 | 0.829 | 0.910 | 0.940 | 0.756 | 0.853 | 0.924 | 0.951 |
| Φ | | 0.467 | 0.599 | 0.720 | 0.772 | 0.587 | 0.713 | 0.819 | 0.861 | 0.641 | 0.762 | 0.858 | 0.896 | 0.673 | 0.788 | 0.879 | 0.914 |

σ: Variance component, n_o_': number of occasions, n_i_': number of items, σ̂^2^_δ_: relative estimated total variance, σ̂^2^_Δ_: absolute estimated total variance, E*ρ*^2^: relative reliability coefficient, Φ: Phi-coefficient (absolute reliability coefficient)

| **Supplementary Table 2.** Path analysis of passing VSAQ, VSAQ understanding levels, and pharmacological MCQ scores | | | | | | | | | | | | |
| --- | --- | --- | --- | --- | --- | --- | --- | --- | --- | --- | --- | --- |
| **Independent variable** | **Pass VSAQ 2** | | | **VSAQ 1 understanding** | | | **VSAQ 2 understanding** | | | **MCQ score** | | |
| **Dependant variable** | **TE** | **DE** | **IE** | **TE** | **DE** | **IE** | **TE** | **DE** | **IE** | **TE** | **DE** | **IE** |
| Pass VSAQ 1 | 0.39 | 0.30* | 0.09 | 0.50 | 0.50* | - | 0.39 | - | 0.39 | 0.02 | - | 0.02 |
| Pass VSAQ 2 | - | - | - | - | - | - |  | 0.37* | - | -0.05 | -0.14 | 0.09 |
| VSAQ 1 understanding | 0.18 | 0.18 | - | - | - | - | 0.63 | 0.56* | 0.07 | 0.13 | - | 0.13 |
| VSAQ 2 understanding | - | - | - | - | - | - | - | - | - | 0.25 | 0.25* | - |
| **R-squared** | 0.17 | | | 0.26 | | | 0.58 | | | 0.04 | | |
| χ²/df= 0.31, CFI= 1.00, TLI= 1.05, RMSEA= 0.01, SRMR= 0.02 | | | | | | | | | | | | |
| TE= Total effect, DE= Direct effect, IE= Indirect Effect, **p*<0.05 | | | | | | | | | | | | |

**Supplementary Figure 2.** The relationship between passing ISF-VSAQs, understanding level groups, and passing the final MCQ exam.


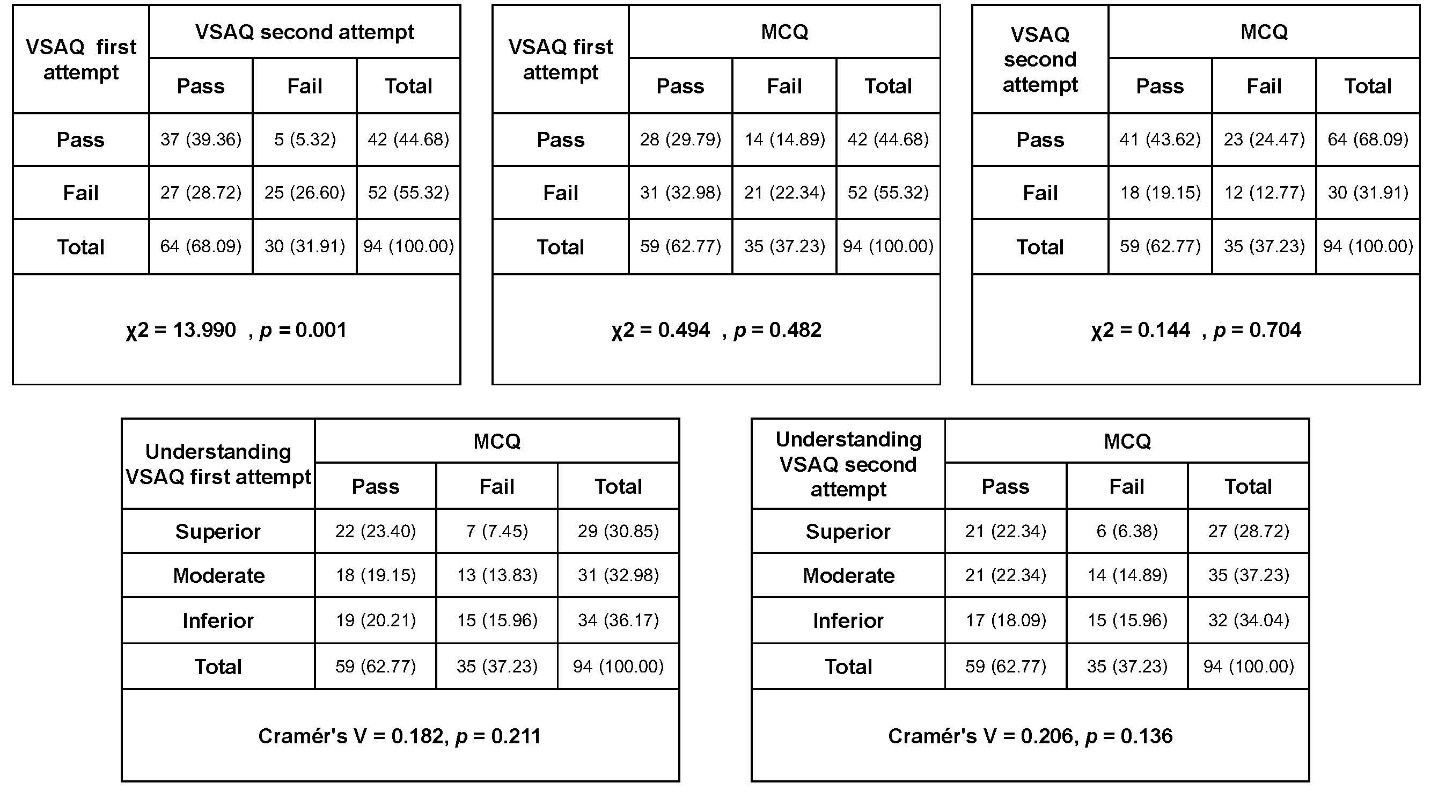

Supplement: Supplementary file 1 — Supplementary Material 1. [file 12909_2024_5569_MOESM1_ESM.docx]
